# A Protocol to Understand the Implementation and Experiences of an Online Community-Based Performance Arts Programme Through and Beyond the COVID-19 Pandemic, *Brain Waves*

**DOI:** 10.3389/fresc.2022.793901

**Published:** 2022-04-15

**Authors:** Carolina Estevao, Elizabeth Taylor, Lucinda Jarrett, Joseph Fort, Kevin Murphy, Anthony Woods, Nikki Crane, Daisy Fancourt, Carmine M. Pariante, Fiona Jones

**Affiliations:** ^1^Department of Psychological Medicine, Institute of Psychiatry, Psychology and Neuroscience, King's College London, London, United Kingdom; ^2^Faculty of Health, Social Care and Education, Centre for Allied Health, St. George's University of London, London, United Kingdom; ^3^Rosetta Life Head Office, Chipping Norton, United Kingdom; ^4^Music Department, King's College London, London, United Kingdom; ^5^Wall2Wall Music C.I.C., Londonderry, Ireland; ^6^Culture Team, King's College London, London, United Kingdom; ^7^Department of Behavioural Science and Health, University College London, London, United Kingdom; ^8^Faculty of Health, Social Care and Education, Centre for Health and Social Care Research, Kingston University and St. George's University of London, London, United Kingdom

**Keywords:** rehabilitation, stroke, acquired brain injured (ABI), community, performance arts

## Abstract

**Introduction:**

Individuals living with acquired brain injury experience numerous psychological, physical, and social challenges. Since the COVID-19 pandemic, many have experienced additional isolation, mental health issues and have had limited access to social and physical activities otherwise available in the community.

**Materials and Methods:**

*Brain Waves* is a 12-week online performance arts programme developed during the COVID-19 pandemic, for people with acquired brain injury (ABI). The research component of *Brain Waves* is a qualitative study, using Interpretative Phenomenological Analysis (IPA) and ethnographic methods (Observations and Interviews). The study will recruit two distinct populations: individuals living with acquired brain injury (including people who have experienced traumatic brain injury and stroke who are participating in the programme) and stakeholders (facilitators, involved in the delivery of *Brain Waves*). This paper presents the protocol for a project which aims to gain an understanding of the implementation and experiences of creating and participating in an online community-based performance arts programme.

## Introduction

Acquired brain injury (ABI) is an umbrella term for any injury to the brain sustained since birth, the most common forms of ABI are traumatic brain injury (TBI; otherwise referred to as head injury) and stroke (1). Individuals with ABI can experience temporary or permanent cognitive, physical and communication impairments. Alongside these impairments, individuals may experience profound challenges in their everyday lives, from medical and social challenges, as well as isolation and loss of independence (2). As a result, a considerable number of individuals experience mental health issues, such as anxiety and depression (3), and even when clinically their injuries are categorized as “mild,” individuals can go on to experience longer-term cognitive, psychological, emotional and social effects, frequently resulting in “hidden disability” (4).

In 2016–2017, in the United Kingdom (UK), there was the equivalent of one hospital admission every 90 s for acquired head injury and the equivalent of one admission every 4 mins for stroke (1). After hospital discharge, there are limited resources for long term recovery and rehabilitation, and clear evidence of the need for greater social, emotional and physical care (5). In addition to individuals experiencing head injury, there are over 1.2 million stroke survivors in the UK, with the numbers expected to rise in the coming decades (6). A recent survey by the UK Stroke Association revealed that 50% of stroke survivors and 85% of carers felt they needed increased support (7). Studies have also shown that in the longer term, individuals with stroke and brain injury are more likely to experience social isolation and a reduction in social networks (8).

Interventions to address social isolation and emotional wellbeing have shown the benefits of engagement in leisure activities, including music and dance. A recent report by Fancourt and Finn (9) found a relationship between engagement in leisure activities and the prevention and management of mental and physical health conditions. To date, over 600 mechanisms of action have been identified underlying these effects, which highlights the complexity and range of interventions and the intentions behind them (10).

For patients with ABI, arts interventions such as music, drama, dance, or painting can be included as part of statutory rehabilitation provision. Making music, including singing and dancing, has been shown to influence mood, emotions, and communication ability (11). Music listening has also been strongly associated with improved mood, memory reminiscence and increased activity in people recovering from a stroke (12). In addition, post-stroke dance programmes have shown a range of benefits, including high levels of participant satisfaction and self-reported improvements in walking and balance (13–15).

The COVID-19 pandemic and subsequent lockdowns have magnified the challenges that individuals with complex conditions face, with the shutdown of services during lockdowns reducing access to rehabilitation and leading to increased isolation and loneliness (16). Research carried out by the UK charity Headway found that more than half (57%) of those who sustained their brain injury within the past 2 years reported that COVID-19 has negatively impacted their access to specialist treatment. A further 64% of those living with the long-term effects of brain injury reported a deterioration in their mental health due to measures implemented to control the spread of COVID-19. Subsequently, almost two thirds (62%) say they now fear for their future (17).

A recent review by King's College London researchers has shown that social isolation in response to public health crises has the greatest mental health impact on the vulnerable and the disadvantaged. The study reviewed 50 papers and found that following periods of isolation either at an individual or community level in the context of public health crises, mental health problems are more common in vulnerable and disadvantaged groups (18). This impact was most prominent for people whose vulnerability was multifaceted, for example, those on a low income, with other co-morbidities or of an ethnic minority background.

Considering the extent of social isolation measures in the current COVID-19 pandemic, the current situation calls for more research targeted at specific groups to understand their experience at this time and the need to develop measures to address the mental health impacts of the pandemic.

### The Impacts of COVID-19 Pandemic on Cultural and Artistic Activities for People With ABI

The COVID-19 crisis has led to a shift in the way in which people access culture, mirrored by the way relevant organizations make cultural activities happen. For people with ABI, these challenges are more acute given their greater isolation and loneliness. Cultural and artistic activities have been re-designed to be delivered online; the same trend has been followed clinically with many therapies now being delivered online or over the phone. Although there is a growing body of evidence supporting the benefits of the arts for health (9), the online delivery of performance arts programmes for people recovering from ABI is novel and has not been researched previously. There are unanswered questions about whether people with ABI can access and engage in online sessions, whether the benefits of programmes can still be achieved when it is delivered online, and if it is possible to build a sense of connection and community between online participants. The benefits of engagement opportunities and the sense of ‘personal community' for people living with long-term conditions has been highlighted by researchers exploring the concept of collective efficacy (20), but whether a personal community can be achieved through online groups is largely unknown. Understanding of how to enable effective participation by those who are not able to access a face to face workshops could also inform new ways of creating personal communities when COVID restrictions no longer apply, and learning about the barriers and facilitators to online delivery of such programmes will help inform future delivery.

### The Work of Rosetta Life for People With ABI

*Rosetta Life* is a UK based charity that has worked with stroke survivors for over two decades and delivers *Brain Odysseys*, a performance arts programme. The programme works on movement skills, music, song, and the spoken word to connect with and build confidence in people living with ABI. *Brain Odysseys* consists of three stages; sessions delivered weekly over 12 weeks; followed by ambassador training (participants who have been through the programme who go onto mentor and support new participants with ABI as Ambassadors for the programme); and a tour of performances in hospitals, community centers and care homes, where new participants for the programme can be recruited (19). Cultural activities, such as those provided by *Brain Odysseys*, also enable individuals with ABI to access social support and new types of creative activities.

### Brain Waves

The “*Brain Waves*” research project was created as part of the COVID-19 pandemic response, uniquely offering a new immersive online cultural programme for individuals with ABI (21). *Brain Waves* will explore the experiences of individuals with ABI accessing cultural activities during the COVID-19 pandemic, as well as exploring the experiences of facilitators delivering the intervention. This project will inform the understanding and implementability of *Brain Waves* and other online performance arts programmes, and the feasibility of adoption and spread to other locations. Additionally, findings will help inform other arts and health organizations to develop and deliver online cultural activities.

This protocol paper will describe the research methods, planned data collection and analysis for this study that is being undertaken.

## Research Methods

### Research Aim and Objectives

The main research aim of this study is to understand the implementation and experiences of an online community-based performance arts programme, *Brain Waves*.

The study objectives are as follows:

1. To understand the experiences, benefits, and opportunities of participation in online performance programmes for participants that are living with acquired brain injury.
2. To study the mechanisms of delivery and interactions between participants and facilitators which take place during online delivery and the concept of creating a personal “community” in the new context of an online group.
3. To use the learning and experiences of facilitators and participants to inform the development of online training to scale up opportunities for online access to arts and culture.
4. To evaluate to what extent the online programme is acceptable, appropriate, and feasible, to participants and stakeholders.
5. To explore how performances by the online community influence attitudes to neurological disability (including attitudes of participants, supporters, and wider stakeholders). This will inform broader discourse and future directions regarding the arts as a vehicle for social change.

### Study Limitations

There are unique limitations in a study conducted in the constantly changing environment of the COVID-19 pandemic. The following limitations have been identified for *Brain Waves:*

a) The project is taking place in an unpredictable and changing context because of ongoing COVID-19 restrictions. As for advice on social distancing and shielding changes, this may make potential participants less eager to participate in an online programme, although we will not be able to know this in advance.
b) Reflexivity and the role of the researcher in the project is also a potential limitation and there is a need to critically reflect on any taken-for-granted assumptions that might influence data analysis and interpretation of findings. The two researchers responsible for all data collection (ET and FJ) are not part of the Brainwaves delivery team, but both come from a clinical and research background in stroke and qualitative methods and may inevitably shape the data generated. Both researchers have critically reflected on the impact of their gender and social status relative to those interviewed – but also need to critically reflect on the need for emerging findings to avoid the potential for affirmation bias when there are many parties invested in the success of the programme. This potential limitation is being addressed by capturing the views of people who have both participated and dropped out of sessions.
c) Due to the nature of this study, there is a symbiosis between the research team at Rosetta Life and the stakeholders of *Brain Waves*. The research team at King's College London has considered this possibility and has mitigated against any bias concerns that may arise during the data collection and analysis. The risk of bias has been mitigated by ensuring that data collection and analysis is conducted by researchers outside of the *Brain Waves* delivery team.

### Study Setting

The 12-week online community-based performance arts programme will be live-streamed to participants from a studio where artists will facilitate each of the 12 sessions. Participants with ABI will watch, listen, and participate remotely on their own or with the support of carers. Observations will be carried out during the sessions by qualitative researchers that will be present in the livestream but not participating.

All interviews with ABI participants and the wider stakeholder group will be conducted online.

The workshops developed online will then be delivered by artists in other locations.

### Intervention Description

In cycle 1, the 12-week online community-based performance arts programme will be facilitated by artists who will be together in a studio and stream a webcast to participants with brain injury, who will be watching, listening and participating in their own homes or another setting of their choice. Together they will create a series of online resources that will be used to support delivery in other locations, enabling wider access to more brain injury survivors.

In cycle 2, the online resources created in cycle 1 will be delivered by a group of local artists. The first three sessions (out of the 12-weekly workshop sessions) of the programme will be delivered online but after that date, there will be a blended approach, some sessions will be delivered in a live space and some sessions online. Participants will be able to choose whether they wish to join in person or online from a setting of their choice.

See the Supplementary Material for a complete template for intervention description and replication (TIDieR) checklist and guide (23) of *Brain Waves*.

### Study Flowchart

**Figure 1:**
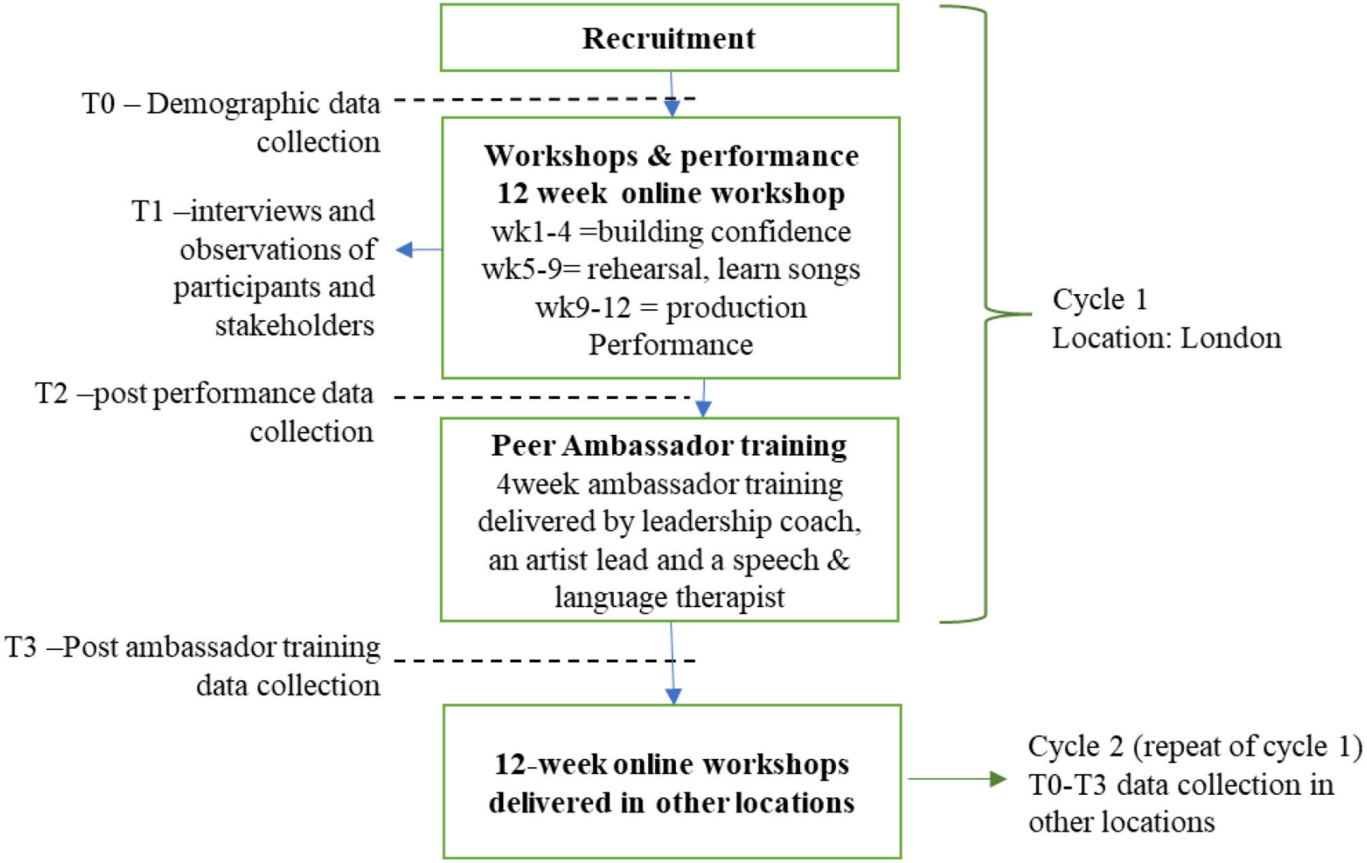
*Brain Waves* Flowchart (cycles 1 and 2). Data collection timepoints at T0, T1, T2 and T3 of both cycles. First cycle (recruitment, workshops and performance and peer ambassador training will be conducted in London); cycle 2 will be conducted in other locations and the same data collection schedule will take place.

The flowchart of the study, that comprises of cycles 1 and 2, can be seen below (Figure 1).

### Sample and Recruitment

We aim to recruit up to 20 ABI participants and 20 stakeholders for this qualitative study. This is an estimate based on the number of participants with ABI previously delivered by Brain Odysseys as in-person programmes and adjusted for the duration of this project (12 months).

The online performance art sessions delivered by Rosetta Life have been communicated to individuals with ABI by signposting in community centers, care homes and the engagement offered through the presentations, screenings, and taster sessions. Stakeholders (artists, ambassadors, family members, technical supporters) will be recruited from the network of people involved in the delivery facilitation or support of the programme.

#### Inclusion Criteria for Interviews and Observations

Consenting participants will be included if they are over 18 years of age, have experienced ABI including ABI or had one or more stroke(s) and can follow a 2-stage command and hold a conversation in English if no supporter/friend is available to translate or support communication.

In addition to the ABI participants that enroll on the online programme, we invite all members of a wider stakeholder group involved in the referral, support, and delivery of the programme. Individuals will be included in the study if they are over 18 years of age and can hold a conversation in English if no supporter/friend is available to translate.

#### Exclusion Criteria for Interviews and Observations

Participants will not be included in the study if they have any co-morbidities that prevent participation in online group activities (e.g., dementia or deteriorating or fluctuating palliative conditions), are unable to understand English or are unable to commit to the 12-week programme.

Aphasia, common in this population, will not be an exclusion criterion unless any potential participants lack the capacity to consent. For aphasia only participants, that have capacity to consent but have speech impairments due to brain injury, if necessary, researchers will gain consent through other forms of communication (e.g., gestures, sounds) and reading or writing.

The wider stakeholder group will not be recruited if they are unable to understand English if no supporter/friend is available to translate.

### Methodological Design

The methodological design of this qualitative study is based on Interpretative Phenomenological Analysis (IPA) and ethnographic methods (observations and Interviews).

The philosophical background to IPA is phenomenology and hermeneutics, and is concerned with examining personal lived experience, the meaning of experience to the participant and how participants make sense of that experience (7). IPA will inform both data collection and analysis (7), and focus on a detailed examination of participants' experiences, what those experiences mean to the participants, and how the participants make sense of those experiences (ibid). IPA will inform the interview questions by seeking to examine individuals' experiences in detail. Research (interviews and observations) will seek to understand the experiences of people with ABI participating in a new online iteration of the more established in-person performing arts programme, and the meaning and sense-making of this experience for participants. In addition, the perspective of other stakeholders including those involved in supporting, delivering, and facilitating sessions will be explored, such as artists, ambassadors, family supporters and technical supporters.

Ethnographic methods will be used to study social interactions, behaviors and perceptions that occur between all participants within the online community created for Brainwaves. These methods will include joining the online sessions to observe them, writing ethnographic fieldnotes based on these observations, and conducting semi-structured interviews with participants and artists. The aim is to gain holistic insights into people's views and actions, as well as the nature of the location (context) they inhabit. The aim has been described as “getting inside” the way each group of participants sees the world through the online interface (22). Ethnography has a strong emphasis on ‘unstructured data' and involves implicit interpretation of the meaning and function of human interactions, rather than hypothesis testing.

## Data Collection

1) Data collection will include ethnographic observations and semi-structured interviews, all collected remotely using Zoom.
2) Observations of the sessions will capture the dynamics of facilitator and participant interactions, practices, and routines. The observations will last for the duration of each session (up to 2 hours) and the researcher present will collect field notes contemporaneously following consent from the group. The researchers will be introduced at the start of each session and the purpose of their presence and request to observe will be requested from all participants. Data will be in the form of written field notes taken during the sessions that describe interactions between participants and facilitators and observation of their verbal and non-verbal responses.
3) Semi-structured interviews with participants and artists involved in designing and delivering the programme, will be carried out remotely using Zoom and based on a topic guide developed with the study team.

Interviews will be audio-recorded and transcribed before analysis, and pen and paper will be used for jotting down observations during the interviews (24). All participants will be free to opt out of any of the research activities and/or withdraw from the research altogether without having to give a reason. If a participant is not willing for researchers to include their data in the observations, researchers will ensure that no data will be recorded about them.

## Data Analysis

Interviews will be transcribed verbatim and read at least twice to get a sense of the essence of lived experiences described by participants. The initial data coding stages will involve making notes in the margins of the transcripts reflective of the language used by participants, condensing participants' responses to a manageable format. ‘Meaning units' or themes will be then derived by the researchers capturing the essence of the lived experience in categories. Field notes were recorded during each observation, written in full immediately afterwards and summary memos produced. Data will be subject to the same thematic analysis used for interview data.

In keeping with the phenomenological philosophical approach, researchers will ‘bracket' their own experience before analyzing interview data, by describing their own experience of the programme. This will involve reading and re-reading observational fieldnotes and reflections. The aim of this is to keep the researchers' experiences distinct from those of the participants. The relationship of the researchers with the research context they are investigating will be presented in the form of a written narrative of ideas and experiences during data collection. These reflective summaries will be shared with the research team and externally to judge any possible biases with the way the data was collected or prior assumptions. Member checking will be carried out with participants and emerging findings will be shared to provide them with an opportunity to verify whether the findings reflect their experiences.

## Anticipated Outcomes and Impact

The table below lists the study objectives (set out in Section Research Aim and Objectives) and their anticipated outcomes and impact. Outputs will inform future directions in the field of online arts delivery for people with ABI (Table 1).

**Table 1:** Objectives and anticipated outcomes and impacts.

**Objective**	**Anticipated outcomes and impacts**
• To explore to what extent the online programme is acceptable, appropriate and feasible to participants and artists/facilitators	Disseminating useful learning including training resources regarding the delivery of online workshops for people with ABI, including whether the online format is more accessible for people with certain characteristics e.g., relating to impairment or level of disability; how long people can engage for online; optimal structuring of content and breaks; strategies to support facilitators to deliver effectively online
• To understand the experiences of participation in the online programme, including benefits and challenges, and whether it is possible to develop a sense of community participation online.	Informing the evidence base regarding the value of online arts for health workshops; creating training materials including potential barriers and facilitators to successful programmes; to develop theoretical insights into the potential of remote participation for forming collective efficacy, meaningful connections and community.
• To study the impact and influence of contextual factors and mechanisms of delivery of the online programme	Informing a logic model to help us a further theoretical understanding of the inputs needed to achieve the desired outcomes of online programmes such as Brainwaves
• Exploring similarities and differences in the delivery of the online programme across different settings, to inform sustainability and spread beyond the project	Disseminating a new understanding of the core components, flexibility and scope for adaption in different settings will inform the development and implementation of similar programmes across different regions, countries and continents.

## Risk Mitigation

Participants with ABI may have physical, psychological, emotional, and social needs. Additionally, participants may be experiencing anxiety and depression because of increased social isolation.

We have instigated a series of checkpoints within the online sessions to guarantee that safeguarding is monitored, and distress can be handled appropriately. If people become distressed participants are offered support offline and personal phone calls. Next of kin details will be collected so that any concerns can be relayed with the consent of participants.

All ABI participants will be supported by experienced artists, ambassadors, and support staff, that will be present, from the consent process, through to the online sessions and screenings.

## Peer Review

The study has been reviewed externally as part of a grant application to the Arts and Humanities Research Council (AHRC) that funds this study.

The protocol has been further reviewed and developed by the programme stakeholders (including artists from Rosetta Life and researchers from St Georges University of London and Kingston University), healthcare professionals, artists and patient and public representatives. In addition, the protocol has been reviewed internally by experts from King's College London who have the requisite knowledge of the clinical and service aspects of the protocol as well as the methodological aspects of the study.

## Patient and Public Involvement

The Brainwaves team have engaged with the ABI community through bi-monthly updates in the form of an online newsletter. To ensure continued dialogue between the different parts of the study, the research team will attend meetings with both Rosetta Life staff and ambassadors at three-time points during the study to share preliminary analysis and gain feedback about any emerging questions or issues.

## Discussion

*Brain Waves* is to our knowledge the only online performance arts study that brings together artists and ABI survivors tailored to the challenges of the COVID-19 pandemic. This study will contribute to the knowledge and understanding of developing and participating in online performance arts for individuals living with ABI. Given the high incidence of social isolation in this group, exacerbated by the COVID-19 pandemic, this project will facilitate understanding of both the practical aspects (challenges and solutions) of how participants have accessed the online community, as well as the constructions of their role and that of their peers and support community.

We anticipate the findings can be used to inform how the programme can be applied across other areas, the level of technical know-how required, support systems and how the benefits can be communicated to the wider population of individuals living with ABI.

## Outputs and Dissemination

The main dissemination activities for *Brain Waves* will be:

1. To widely disseminate resources, frameworks and research findings across Arts Higher, Education Institutions, NHS Commissioning Bodies and Arts and Healthcare Organizations.
2. To publish a research report online outlining the learning from the perspectives of the arts/creativity, healthcare and community engagement, together with relevant videos.

The outputs of *Brain Waves* are to create:

1. A research report publishing findings and, implications in relation to future directions for arts, healthcare and community engagement practice.
2. Publications in peer-reviewed journals.
3. A learning package of resources for artists so that *Brain Waves* can be replicated in other locations.

## Data Availability Statement

The original contributions presented in the study are included in the article/Supplementary Material, further inquiries can be directed to the corresponding author/s.

## Ethics Statement

Ethical approval has been granted by the King's College London PNM Research Ethics Panel, REC Reference: HR/DP-20/21-22443.

## Author Contributions

CE, ET, and FJ: conceptualization, methodology, and project administration. LJ, NC, AW, DF, and CP: conceptualization, project administration, and funding acquisition. KM and JF: conceptualization and methodology. All authors have been involved in the drafting of the manuscript and have individually approved the version of the work published.

## Funding

This study is funded by the Arts and Humanities Research Council (AHRC) UKRI Ideas to Address COVID-19 [Award AH/V014870/1]; the study sponsor is King's College London.

## Conflict of Interest

CP reports Grants from Wellcome Trust, during the conduct of the study; Grants from National Institute for Health Research (NIHR), Grants from NIHR Senior Investigator, grants from Johnson and Johnson, grants from Wellcome Trust, outside the submitted work. LJ was employed by Rosetta Life. KM was employed by Wall2Wall Music C.I.C. The remaining authors declare that the research was conducted in the absence of any commercial or financial relationships that could be construed as a potential conflict of interest.

## Publisher's Note

All claims expressed in this article are solely those of the authors and do not necessarily represent those of their affiliated organizations, or those of the publisher, the editors and the reviewers. Any product that may be evaluated in this article, or claim that may be made by its manufacturer, is not guaranteed or endorsed by the publisher.
